# Adipose tissue loss during neoadjuvant chemotherapy: a key prognostic factor in advanced epithelial ovarian cancer

**DOI:** 10.3389/fphys.2025.1537484

**Published:** 2025-03-25

**Authors:** Wassim Benouali, Adeline Dolly, Aurore Bleuzen, Stéphane Servais, Jean-François Dumas, Christophe Vandier, Caroline Goupille, Lobna Ouldamer

**Affiliations:** ^1^ Radiology Department, Hôpital Bretonneau, CHRU de Tours, Tours, France; ^2^ Laboratoire N2Cox, INSERM U1069, Université de Tours, Tours, France; ^3^ Gynecology Department, Hôpital Bretonneau, CHRU de Tours, Tours, France

**Keywords:** epithelial ovarian cancer, body composition, neoadjuvant chemotherapy, ovarian cancer, sarcopenia, visceral adipose tissue

## Abstract

**Background:**

Advanced epithelial ovarian cancer (EOC) patients often receive neoadjuvant platinum-based chemotherapy (NAC), with interval surgery (after three cycles of chemotherapy) considered as a major prognostic factors. We examined how changes in body composition (muscle and adipose tissue) during NAC influence prognosis.

**Objective:**

Using CT images acquired before and during NAC in a cohort of women with advanced EOC, the aim of this study was to analyze body composition (muscle and fat mass) and see whether these parameters, at diagnosis or as they evolve during chemotherapy, can be linked to recurrence-free survival and overall survival (RFS and OS).

**Material and methods:**

The study included 53 patients with FIGO stage III-IV epithelial ovarian cancer. CT images were analyzed to calculate skeletal muscle index (SMI), subcutaneous adipose tissue index visceral adipose tissue index estimated lean body mass (LBM) and estimated whole-body fat mass (WFM). Changes in tissue composition were normalized for 100 days and expressed as % change to account for intervals between scans at baseline and after three cycles of chemotherapy. The impact on survival was assessed by Log-rank test.

**Results:**

At diagnosis, clinical criteria such as age or BMI did not correlate with RFS or OS. 60% of patients were considered sarcopenic (low SMI), including mainly underweight and normal-weight patients. Low SMI was not associated with RFS or OS. Twenty-six patients who underwent interval surgery demonstrated longer relapse-free intervals (*p* = 0.01). Notably, while muscle parameters showed minimal changes (−2%), parameters assessing adipose tissue showed significant decreases of 10, 12% and 7.6% per 100 days (VATI, SATI and estimated WFM, respectively). Obese patients were particularly affected by this loss of muscle and fat, compared with patients in other BMI categories. Rapid and severe loss of VATI (−28% per 100 days) and estimated WFM (−18% per 100 days) were significantly associated with shorter OS (*p* = 0.031 and *p* = 0.046 respectively).

**Conclusion:**

Our findings suggests that early and substantial loss of visceral adipose tissue during NAC is a significant predictor of poor survival in advanced EOC. This highlights an urgent need for targeted nutritional or pharmaceutical strategies to mitigate fat loss and improve patients outcomes.

## Introduction

In addition to new targeted therapies (anti-angiogenic and PARP inhibitors), the treatment of epithelial ovarian cancer consists of primary excision surgery followed by chemotherapy (Armstrong et al., 2021). However, in patients with advanced disease, platinum-based neoadjuvant chemotherapy has been introduced as an option to decrease tumor burden and increase the optimal cytoreduction rate for complete surgery. Excisional surgery is then performed, if possible, after three or four cycles of chemotherapy, known as interval surgery. The International Federation of Gynecology and Obstetrics (FIGO) stage and residual disease after surgery remain well-recognized factors in survival prognosis (Hennessy et al., 2009), and NAC helps reduce the risk of early mortality (Melamed et al., 2021). However, epithelial ovarian cancer (EOC) is often diagnosed at an advanced stage, and the overall 5-year survival rate remains very low, at around 50% (Melamed et al., 2021; Siegel et al., 2018). These high mortality rates underline the need for new therapeutic strategies (Nikolaidi et al., 2022), and the search for markers associated with relapse or survival can help identify patients with the poorest prognosis, promote individual management and guide the search for new therapeutic targets.

Used as a standard method of managing cancer staging and follow-up, CT imaging can also provide a more accurate analysis of body composition than weight or BMI alone. Using thresholds commonly used in the literature (Martin et al., 2013a), muscle and fat areas can be quantified. After normalization for patient height, the SMI (Skeletal Muscle Index), ATI (Adipose Tissue Index), estimated LBM (Lean Body Mass) and estimated WFM (Whole-Body Fat mass) can be calculated (Shen et al., 2004; Mourtzakis et al., 2008a; Prado et al., 2008a). Clinically, the diagnosis of cancer-related sarcopenia (loss of muscle mass and functionality) may go undetected if weight loss is masked by ascites accumulation or adiposity. CT analysis provides a more precise and specific picture of body composition.

Numerous studies have attempted to identify body composition parameters at baseline (cancer diagnosis), associated with relapse and survival. A low muscular index has already been associated with poorer survival, as in pancreatic (Klassen et al., 2023) and colorectal (Hopkins et al., 2019) cancers. In ovarian cancer, the meta-analysis by Ubachs *et al.* (Ubachs et al., 2019) showed a significant association between low SMI and overall survival, while McSharry *et al.* identified only a link between muscle attenuation and survival (McSharry et al., 2020). Recently, the meta-analysis by Jin *et al.* (Jin et al., 2023) showed that low SMI is associated with progression-free survival, 5-year overall survival and highlighted its association with advanced FIGO stage and low BMI (<25 kg/m^2^) but not with histological types or chemotherapy toxicity.

Several studies show that this body composition can change progressively with muscle and/or fat loss during cancer follow-up. The origins and kinetics of these losses remain unclear (Klassen et al., 2023; Vazeille et al., 2017; Cuello et al., 2023). Associations between muscle and fat loss and outcome have been demonstrated in patients with foregut cancers, for example, (Daly et al., 2018). In a tumor-bearing rat model, adipose tissue loss and the onset of lipolysis occur before muscle loss and reduced food intake (Byerley et al., 2010). In cancer patients, weight loss is also associated with lipolysis, and the kinetics of adipose tissue loss appear to be greater than those of lean body mass. This fat loss is also accompanied by changes in circulating lipids (Murphy et al., 2010; Fouladiun et al., 2005). This lipolysis was also observed in our previous study of ovarian cancer patients. Loss of n-6 and n-3 polyunsaturated fatty acids in adipose tissue was associated with earlier relapse (Salaun et al., 2023).

While several studies suggest the importance of fatty acid metabolism in ovarian cancer progression (Cai et al., 2015; Ladanyi et al., 2018; Mukherjee et al., 2020), few clinical studies with CT analysis report the amount of adipose tissue at baseline, its evolution during treatment and its prognostic value. In 2013, Torres *et al.* showed that low amounts of subcutaneous adipose tissue (SAT) and intermuscular adipose tissue (IMAT) at diagnosis were associated with poorer overall survival (Torres et al., 2013). In 2022, Nakayama *et al.* concluded that fat loss was not associated with disease-free survival and overall survival (Nakayama et al., 2022). Huang *et al.* reported that muscle loss but not fat loss was associated with poor survival in a homogeneous population of stage III ovarian cancer patients (Huang et al., 2020). The latter two studies excluded patients for whom NAC was indicated. Only Rutten *et al.* in 2016 reported that loss of visceral adipose tissue and muscle during neoadjuvant chemotherapy negatively influenced overall survival (Rutten et al., 2016).

Given the limited amount of research in this specific area of NAC in ovarian cancer patients, we sought to assess body composition parameters (skeletal muscle and adipose tissue), at baseline and their evolution, in a cohort of women treated with NAC for advanced epithelial ovarian cancer and to determine whether these parameters or their changes during the first cycles of chemotherapy have prognostic value.

## Patients and methods

### Study design and patients

**FIGURE 1:**
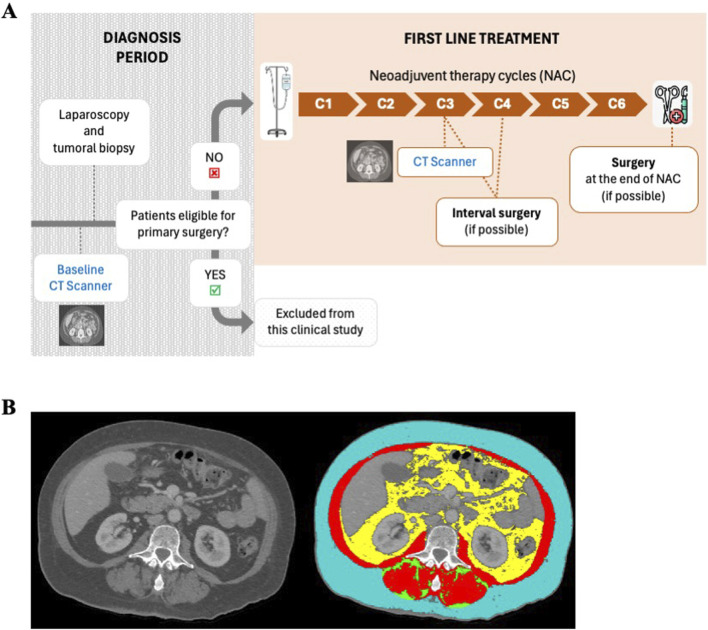
Care pathway and CT scans analysis of ovarian cancer patients included in our retrospective clinical study. **(A)** During the diagnostic period, CT scan (defined as the reference CT scan) and laparoscopy were performed to allow staging of the cancer, definition of tumor histology and operability. When not eligible for primary surgery (unsatisfactory debulking), patients underwent neoadjuvant platinum-based chemotherapy. After three to four cycles of chemotherapy, further CT imaging (defined as C3-C4 CT scan) was performed to decide whether interval surgery was possible. Fifty-three women with available CT scans (at baseline and C3-C4) were included in this retrospective clinical study. Patients eligible for primary surgery were excluded. **(B)** Body composition analyzed on a patient’s CT scan using SliceOmatic software (Tomovision). Tissues are identified according to their contrast in Hounsfield Units (HU): skeletal muscle in red (−29 to +150 HU), subcutaneous adipose tissue in blue (−190 to −30 HU), intermuscular adipose tissue in green (−190 to −30 HU) and visceral adipose tissue in yellow (−150 to −50 HU).

We collected data on all women with presumed advanced-stage EOC and FIGO 2009 final stage III-IV cancer (Pecorelli, 2009) managed at the University Hospital of Tours from January 2016 to December 2019. The care pathway of patients included in this study is presented in Figure 1A. During the diagnostic period, an extension workup is performed, notably using CT scans and laparoscopic biopsies. CT images acquired during this period are considered the patient’s baseline image (C0). Patients who were not eligible for primary reduction surgery were included in our study and received neoadjuvant chemotherapy (NAC) consisting of carboplatin (AUC 5) and paclitaxel (175 mg/m^2^) every 21 days. Women under 65 years old received a maximum of 750 mg paclitaxel per cycle, while women over 65 years old received a maximum of 600 mg per cycle. Including patients over 65 years old was essential to reflect the real-world demographic of advanced epithelial ovarian cancer (EOC), as older age is a significant factor in both disease prevalence and treatment outcomes. While older age is associated with increased sarcopenia, it also represents a critical subgroup that may experience differential responses to neoadjuvant chemotherapy (NAC) and body composition changes.

Patients eligible for primary surgery were excluded to focus on a homogeneous cohort of advanced EOC patients who required NAC. This exclusion ensures that the study population reflects those with the highest disease burden, for whom NAC is most relevant. While this limits the generalizability of findings to all EOC patients, it strengthens the internal validity of the study by reducing heterogeneity.

After three cycles of NAC, a new CT-scan was performed to assess tumor response and validate the possibility of interval surgery. CT-scan images acquired at this time are identified as C3 images. If patients were still not eligible for full surgery after three or four cycles, NAC was continued for a total of six to eight cycles.

The research protocol using anonymized CT images was approved by the institutional review board.

### CT-scan analyses

We retrieved the abdominopelvic CT scans initially used for cancer diagnosis/follow-up to assess patients’ body composition. An axial image of the third lumbar vertebra (L3) was selected for analysis of total muscle and fat cross-sectional areas (cm^2^) (Mourtzakis et al., 2008b; Prado et al., 2008b; Shen et al., 1985; Mitsiopoulos et al., 1985). Tissues were anatomically identified and quantified in predefined Hounsfield Unit (HU) ranges (Mitsiopoulos et al., 1985): skeletal muscle (−29 to +150 HU); subcutaneous adipose tissue (SAT) (−190 to −30 HU); intermuscular adipose tissue (IMAT) (−190 to −30 HU); visceral adipose tissue (VAT) (−150 to −50 HU), using Slice-O-Matic software (v.6.0; Tomovision, Magog, Canada) (Figure 1B). Measurements were performed by a radiologist, who was blinded to the patients’ treatment status.

Cross-sectional area of total muscle was normalized for stature, and skeletal muscle index (SMI; cm^2^/m^2^) was calculated (Mourtzakis et al., 2008b; Prado et al., 2008b). We used validated cut points for sarcopenia (Martin et al., 2013a): SMI <41 cm^2^/m^2^ for women. Patients with a SMI below this cut-point were considered sarcopenic. Total fat cross-sectional area was calculated as the sum of VAT, SAT and IMAT areas. For lean body mass (LBM; kg) and whole-body fat mass (WFM; kg) calculation, previously validated regression equations were used (Mourtzakis et al., 2008b; Shen et al., 1985).

### Statistical analyses

Statistical analyses were performed using GraphPad Prism software for Windows (v.10.3.1, Boston, Massachusetts, United States). Categorical variables were summarized using frequency counts and percentages, and differences between groups were tested using Chi^2^ or Fisher’s exact tests as appropriate. Continuous variables were summarized using median and interquartile range (IQR). We used nonparametric Wilcoxon-Mann-Whitney tests (with median and interquartile range (IQR)) to compare continuous variables. Outliers were excluded using Grubbs’ test (α = 0.05).

Survival analysis was performed using Kaplan-Meier estimates to estimate the event-time distributions for recurrence-free survival (RFS) and overall survival (OS). Differences between risk groups were compared using the log-rank. Cox proportional hazards models were used to assess the multivariate effect of covariates. Statistical significance was set at a p-value less than 0.05.

We considered a *p*-value ≤0.05 to be statistically significant.

## Results

### Characteristics of the study cohort

Fifty-three women were included in this retrospective clinical study. They received platinum-based neoadjuvant chemotherapy (NAC) for advanced epithelial ovarian cancer (EOC) and had available CT-Scans at baseline and C3 (Figure 1A). Patient characteristics are presented in Table 1. The median age was 68 years, with a wide range from 23 to 84 years. The median BMI was 24.7 kg/m^2^ (Interquartile Range (IQR) 21.9–27.9), with eight obese patients (15%) included in the cohort. Seventeen patients (32%) experienced body weight loss 2–6 months prior to the inclusion. Regarding tumor histology: 43 women (81%) had pure high-grade serous ovarian cancer (HGSOC) and 10 (19%) had EOC types (2 women had HGSOC mixed with undifferentiated type, two had carcinosarcomas, two had endometrioid type and three had clear-cell carcinoma type). Twenty-one (40%) had ascites removed during diagnostic laparoscopy. After three to four cycles of NAC, 49% of patients were eligible for surgery (interval surgery), while 19% could only be operated on after six cycles of chemotherapy, and 32% were not operated on at all, showing that around 50% of patients responded poorly or not at all to chemotherapy.

**TABLE 1:** Patient characteristics (*n* = 53).

Clinical parameters	*n* (%)	Median [IQR]
Age (years)		68 [62.5–73.0]
Menopausal status		
Non menopausal patients	8 (15%)	
BMI (kg/m^2^)		24.7 [21.9–27.9]
Underweight (≤18.5)	3 (6%)	
Normal weight (18.5–24.9)	25 (47%)	
Overweight (25–29.9)	17 (32%)	
Obese (≥30.0)	8 (15%)	
BW loss during the past 2–6 months (%)		7.3 [4.1–10.6]
Patients who experienced BW loss	17 (32%)	
Ascites punction (L)		3.8 [0.9–5.0]
Patients with ascites removed	21 (40%)	
Histology		
Pure HGSOC	43 (81%)	
Other cancer types	10 (19%)	
FIGO Stage		
Stage III	35 (66%)	
Stage IV	14 (26%)	
Unknown	4 (8%)	
Treatment		
Surgery after 3–4 cycles of chemotherapy	26 (49%)	
Surgery after 6 cycles of chemotherapy	10 (19%)	
Not operable at anytime	17 (32%)	
Follow up (months)		13.0 [9.0–22.8]

BMI, body mass index; BW, body weight; FIGO, federation of gynaecology and obstetrics classification for staging ovarian cancer; HGSOC, high grade serous ovarian cancer; IQR, interquartile range; SD, standard deviation.

### Baseline clinical and body composition parameters and outcomes

**FIGURE 2:**
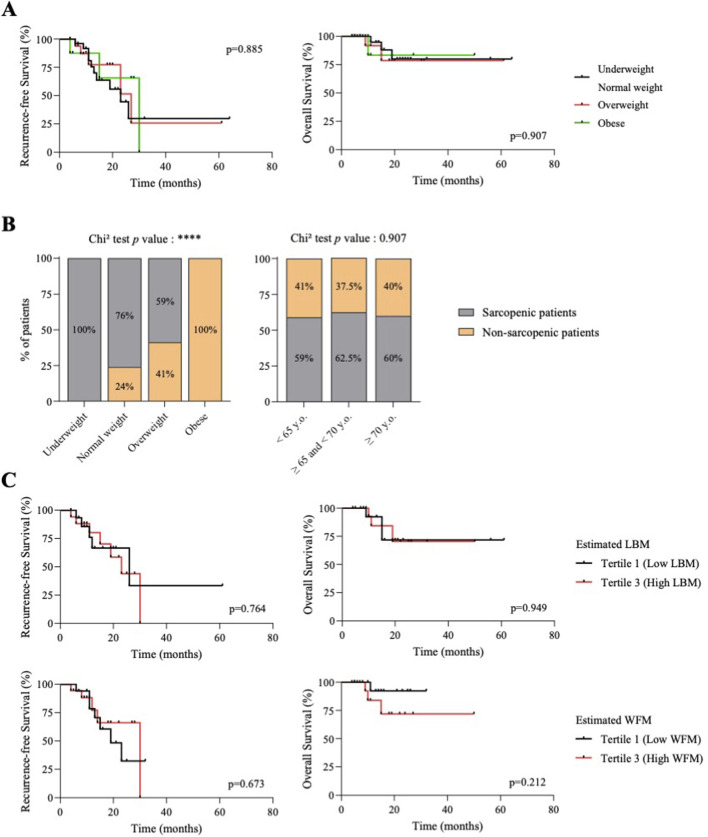
BMI and body composition parameters at baseline do not influence the survival of patients with ovarian cancer. **(A)** Kaplan-Meier curves of recurrence-free survival (left) and overall survival (right). Patients were divided into BMI categories. *P* value were calculated by Log-rank tests. **(B)** Proportion of sarcopenic and non-sarcopenic patients by body mass index category (BMI - left) or age tertiles (right). Chi^2^ tests were used to generate *p* values. **(C)** Kaplan-Meier curves of recurrence-free survival (left) and overall survival (right). Patients were divided according to baseline body composition parameters: lean body mass (LBM) or whole-body fat mass (WFM). Black lines represent patients with low LBM (<34.3 kg, tertile 1) or low WFM (<17.6 kg, tertile 1). Red lines represent patients with high LBM (>38.6 kg, tertile 3) or high WFM (>26.4 kg, tertile 3). *P* value were calculated by Log-rank tests.

We investigated the association between clinical and body composition parameters at baseline (C0) and survival in our cohort. Results are presented in Table 2 and Figure 2. For most parameters, patients were divided into tertiles, and we compared recurrence-free survival (RFS) and overall survival (OS) between tertiles 1 and 3. For BMI, patients were classified as underweight (BMI <18.5 kg/m^2^), normal weight (18.5 ≤ BMI <25 kg/m^2^), overweight (25 ≤ BMI <30 kg/m^2^) and obese (BMI ≥30 kg/m^2^). We also compared sarcopenic and non-sarcopenic patients on the basis of their skeletal muscle index (SMI<41 cm^2^/m^2^), as described in the methods section.

**TABLE 2:** Clinical and body composition parameters at baseline and their association with survival in ovarian cancer patients treated with NAC.

Parameters	Groups	n (%)	Median [IQR]	*p* value for RFS (low vs. High)	*p* value for OS (low vs. High)
Clinical parameters					
Age (years)	All patients	53 (100%)	68.0 [62.5–73.0]	0.245	0.896
	Tertile 1 (Younger)	17 (32%)	51.0 [33.5–62.5]		
	Tertile 3 (Older)	20 (38%)	75.0 [71.0–78.5]		
BMI (kg/m^2^)	All patients	53 (100%)	24.7 [21.9–27.9]	0.885	0.907
	Under/Normal weight	28 (53%)	22.0 [21.0–23.8]		
	Overweight	17 (32%)	27.0 [26.4–28.0]		
	Obese	8 (15%)	32.5 [31.0–37.9]		
Muscle parameters					
SMI (cm^2^/m^2^)	All patients	53 (100%)	39.4 [35.4–44.2]	0.234	0.830
	Sarcopenic patients (Low SMI)	32 (60%)	36.6 [34.1–38.8]		
	Non-sarcopenic patients (High SMI)	21 (40%)	45.4 [43.1–50.6]		
Estimated LBM (kg)	All patients	53 (100%)	37.1 [33.3–40.5]	0.764	0.949
	Tertile 1 (Low LBM)	17 (32%)	32.5 [30.7–33.4]		
	Tertile 3 (High LBM)	18 (34%)	41.1 [40.5–43.0]		
Fat Parameters					
VATI (cm^2^/m^2^)	All patients	53 (100%)	22.5 [11.6–47.3]	0.537	0.164
	Tertile 1 (Low VATI)	18 (34%)	9.0 [2.8–12.5]		
	Tertile 3 (High VATI)	18 (34%)	68.0 [46.3–100.1]		
SATI (cm^2^/m^2^)	All patients	53 (100%)	66.3 [48.0–121.6]	0.793	0.133
	Tertile 1 (Low SATI)	18 (34%)	34.7 [26.0–48.9]		
	Tertile 3 (High SATI)	18 (34%)	125.6 [115.9–157.7]		
Estimated WFM (kg)	All patients	53 (100%)	21.3 [16.0–33.1]	0.673	0.212
	Tertile 1 (Low WFM)	18 (34%)	12.7 [10.1–16.1]		
	Tertile 3 (High WFM)	18 (34%)	36.2 [32.8–42.5]		

BMI, body mass index; IQR, interquartile range; LBM, lean body mass; OS, overall survival; RFS, Recurrence-Free Survival; SATI, subcutaneous adipose tissue index; SMI, skeletal muscle index; VATI, visceral adipose tissue index; WFM, Whole-Body Fat Mass.

Age and BMI had no significant influence on RFS and OS in our cohort (Table 2; Figure 2A). Regarding skeletal muscle parameters, median estimated LBM was 37.1 kg (IQR 33.3–40.5) and median SMI was 39.4 cm^2^/m^2^ (IQR 35.4–44.2). Thirty-two patients (60%) were classified as sarcopenic at diagnosis (C0), with median SMI being 36.6 cm^2^/m^2^ (IQR 34.1–38.8) (Table 2). The proportion of sarcopenic patients differed significantly according to BMI category (*p* < 0.0001) (Figure 2B): 100% of underweight patients, 76% of normal-weight patients and 59% of overweight patients were sarcopenic. The eight obese patients were not sarcopenic. The proportion of sarcopenic patients remained unchanged across all age categories (*p* = 0.90) (Figure 2B). There was no association between estimated LBM or sarcopenic status at baseline and patients survival (RFS and OS) (Table 2; Figure 2C). When looking at adipose tissue parameters at baseline, whether subcutaneous adipose tissue index (SATI), visceral adipose tissue index (VATI) or estimated whole body fat mass (WFM), none were associated with RFS or OS (Table 2; Figure 2C).

Altogether, this first set of results shows that patients’ body composition at baseline (whether muscle or adipose tissue parameters) was not associated with survival (RFS/OS) in our cohort of advanced EOC patients treated with NAC.

### Body composition changes during NAC and outcomes

**FIGURE 3:**
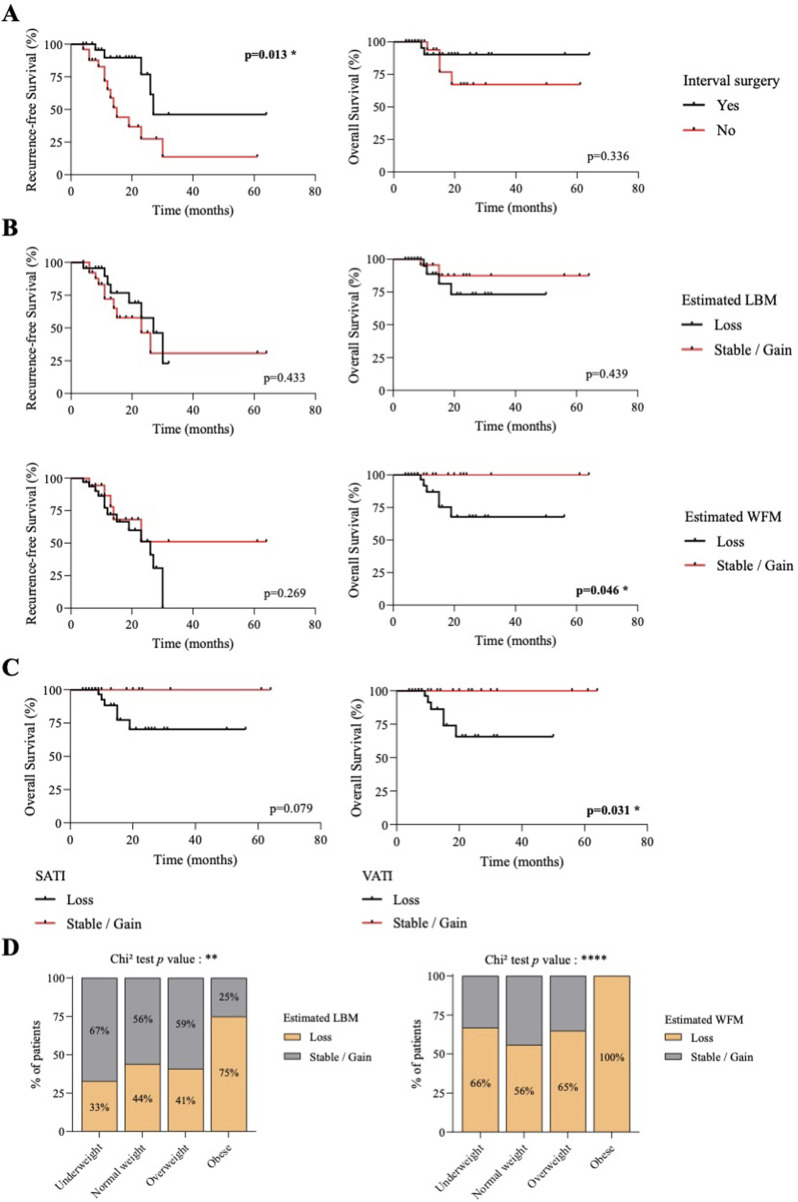
Loss of body fat, but not lean body mass, during neoadjuvant chemotherapy is associated with shorter survival for ovarian cancer patients. **(A)** Kaplan-Meier curves of recurrence-free survival (left) and overall survival (right) according to interval surgery eligibility. Black lines, patients with interval surgery. Red lines, patients without interval surgery. *P* value were calculated by Log-rank tests. **(B)** Kaplan-Meier curves of recurrence-free survival (left) and overall survival (right) according to changes in LBM or WFM during NAC. Black lines, patients who lost LBM or WFM. Red lines, patients who gained or had stable LBM or WFM. *p* value were calculated by Log-rank tests. **(C)** Kaplan-Meier curves of overall survival according to changes in subcutaneous (left) or visceral (right) adipose tissue index (SATI/VATI) during NAC. Black lines, patients who lost SATI or VATI. Red lines, patients who gained or had stable SATI or VATI. *p* value were calculated by Log-rank tests. **(D)** Proportion of patients with loss or gained/stable lean body mass (LBM - left) or whole-body fat mass (WFM - right) during NAC according to BMI category. Chi2 tests were used to generate p values.

Twenty-six women (49%) underwent interval surgery after three cycles of NAC. This suggested a good tumor response. Indeed, patients eligible for interval surgery showed a prolonged time to cancer relapse (median RFS 27 months vs. 15 months for eligible and non-eligible patients respectively - *p* = 0.013), but a survival time close to that of patients who were not eligible (OS - *p* = 0.336) (Table 3; Figure 3A).

**TABLE 3:** Changes in clinical parameters and body composition between C0 and C3 and their association with survival in ovarian cancer patients treated with NAC.

Parameters	Groups	n (%)	Median [IQR]	*p* value for RFS (stable/Gain vs. Loss)	*p* value for OS (stable/Gain vs. Loss)
Clinical Parameters					
Interval surgery at 3–4 cycles	Yes	26 (49%)		0.013 *	0.336
	No	27 (51%)			
Muscle parameters					
SMI (%/100 days)	All patients	53 (100%)	−2.2 [-8.4; +6.5]	0.113	0.645
	SMI Stable/Gain	26 (49%)	+6.5 [+3.0; +9.0]		
	SMI Loss	27 (51%)	−7.8 [-12.8; −4.3]		
Estimated LBM (%/100 days)	All patients	53 (100%)	−1.8 [-6.9; +5.4]	0.433	0.439
	LBM Stable/Gain	28 (53%)	+4.5 [+2.2; +7.6]		
	LBM Loss	25 (47%)	−6.9 [-12.3; −3.9]		
Fat Parameters					
VATI (%/100 days)	All patients	53 (100%)	−10.5 [-32.5; +6.6]	0.249	0.031 *
	VATI Stable/Gain	19 (36%)	+17.2 [+5.0; +39.0]		
	VATI Loss	34 (64%)	−27.7 [-39.6; −10.8]		
SATI (%/100 days)	All patients	53 (100%)	−12.1 [-27.3; +1.4]	0.075	0.079
	SATI Stable/Gain	16 (30%)	+10.3 [+2.4; +28.5]		
	SATI Loss	37 (70%)	−19.0 [-32.5; −10.6]		
Estimated WFM (%/100 days)	All patients	53 (100%)	−7.6 [-20.8; +2.0]	0.269	0.046 *
	WFM Stable/Gain	18 (34%)	+4.9 [+1.4; +19.4]		
	WFM Loss	35 (66%)	−17.0 [-24.7; −7.6]		

C0, at diagnosis (before initiation of NAC); C3, after completion of three–four cycles of NAC (interval); IQR, interquartile range; LBM, lean body mass; OS, overall survival; RFS, Recurrence-Free Survival; SATI, subcutaneous adipose tissue index; SMI, skeletal muscle index; VATI, visceral adipose tissue index; WFM, Whole-Body Fat Mass.

The 53 patients included in this cohort had a second CT-Scan at C3 with a median duration interval of 103 days (Min 55; Max 220). Data for change over time were normalized to 100 days. For muscle and fat parameters, patients who experienced tissue loss greater than the 2% measurement error (MacDonald et al., 2011), were categorized as tissue losers. Other patients were categorized as stable/gaining tissue over time. Tissue changes are detailed in Table 3.

Skeletal muscle loss resulted in two women becoming sarcopenic. In line with these results, SMI remained relatively stable between diagnosis (C0) and C3 chemotherapy cycles, with a median variation of −2.2% (IQR -8.4% to +6.5%) (Table 3), suggesting that these first chemotherapy cycles did not drastically worsen sarcopenia.

We compared patients who lost SMI (−7.8%; IQR -12.8% to −4.3%) or LBM (−6.9%; IQR -12.3% to −3.9%), and those who gained or has stable SMI (+6.5%; IQR +3.0% to +9.0%) or LBM (+4.5%; IQR +2.2% to +7.6%). We found no association with RFS and OS (Table 3; Figure 3B).

Adiposity parameters changed more markedly between C0 and C3, with median losses of −10.5% (IQR -32.5% to +6.6%) for VATI, −12.1% (IQR -27.3% to +1.4%) for SATI and −7.6% (IQR -20.8% to +2.0%) for WFM estimation (Table 3). We observed no significant association between changes in body fat and recurrence-free survival (Table 3; Figure 3B). Only loss of SATI tended to be associated with shorter RFS (*p* = 0.075) (Table 3). On the other hand, loss of body fat seems to be a prognostic factor for OS. We observed that patients had shorter overall survival if they had lost VATI (*p* = 0.031) or WFM (*p* = 0.046). The same tends to apply to SATI (*p* = 0.079) (Table 3; Figures 3B,C). Patients with rapid and severe loss of VATI (−27.7%, IQR -39.6% to −10.8%), SATI (−19.0%, IQR -32.5% to −10.6%) and estimated WFM (−17.0%, IQR -24.7% to −7.6%) within 100 days had shortened overall survival.

During their first three cycles of neoadjuvant chemotherapy, obese patients were particularly affected by tissue loss, whether lean or fat mass (Figure 3D), with 75% and 100% of obese patients losing more than 2% of their estimated lean and fat mass respectively.

Overall, these results show that after three cycles of neoadjuvant chemotherapy, obese patients are more likely to lose muscle mass and fat mass. However, it was patients with rapid and marked fat loss who had a significantly shorter overall survival time.

Multivariate Cox regression analysis demonstrated that visceral fat loss remained an independent prognostic factor for overall survival (HR = 2.45, 95% CI: 1.32–4.56, p = 0.004), after adjusting for age, BMI, and surgical status.

### Characteristics of patients eligible or not eligible to interval surgery at C3

Eligibility for interval surgery suggests a better tumor response to NAC and is known to influence a better prognosis, in particular by increasing time to relapse. We retrospectively examined whether patients eligible for interval surgery had specific clinical and body composition characteristics. We compared clinical and body composition parameters at baseline, as well as changes in body composition parameters during NAC, and found no significant differences between patients eligible and ineligible for interval surgery (Supplementary Table 1). Patients with the best tumor response to NAC had no specific clinical or body composition characteristics.

## Discussion

The aim of our study was to assess body composition parameters in women with advanced epithelial ovarian cancer eligible for NAC, and to determine whether these parameters had an impact on prognosis. Low BMI, but not age, was a primary clinical indicator associated with sarcopenic patients, since 100% of underweight women and 76% of normal-weight women, but none of the obese women, were sarcopenic at baseline. No clinical (age, BMI) or body composition parameters at baseline were associated with recurrence-free survival or overall survival. After three cycles of NAC, 26 patients underwent interval surgery. This indicator of good tumor response was associated with a significantly longer time to relapse, but not with a specific change in body composition. During the first three cycles of NAC, the loss of body fat (around −10%) was more marked than the loss of muscle mass (−2%). Obese patients were the most affected by these losses, with 100% showing a reduction in fat mass and 75% a reduction in muscle mass. Nevertheless, it was the patients who had a severe and rapid loss of VATI (−28% per 100 days) and fat mass (−17% per 100 days) who had a significantly shorter overall survival.

In this study, the most commonly used threshold, SMI<41 cm^2^/m^2^, was chosen as the reference to identify 32 sarcopenic patients (Martin et al., 2013b) (60% of patients). Using the reference supported by the Haute Autorité de Santé (HAS), SMI<38.5 cm^2^/m^2^ (Prado et al., 2008c), only 21 women (40%) should be considered sarcopenic prior to the implementation of NAC. The relevance of the chosen threshold is debatable, as thresholds defined for American populations may not be directly applicable to European or Asian populations. A sensitivity analysis using an alternative threshold (SMI <38.5 cm^2^/m^2^) yielded similar results, confirming the robustness of our findings.

However, analyses using this other threshold or a tertile analysis also failed to establish a link between sarcopenia, low SMI or loss of SMI and survival in our study. Several hypotheses can be put forward: i/the patients we included were first-line patients, whereas several studies have reported a loss of muscle mass during palliative chemotherapy; ii/the loss of muscle mass, with a median variation of −2%, remained low. Sarcopenia is characterized by progressive muscle loss. The time between the two scans (median 103 days) may not be sufficient to show a significant reduction in muscle mass and impact on survival; iii/this pilot study included 53 patients, which may be too small a number to demonstrate the effect of low muscle mass or loss of muscle mass on survival. However, the literature is not yet in complete agreement on the association between sarcopenia and survival in EOC (McSharry et al., 2020; Jin et al., 2023; Huang et al., 2020). As shown by Jin *et al.* (Jin et al., 2023), body composition, and in particular muscle mass, can change with disease stage: a low muscle mass index is more frequently associated with advanced stages (stage III/IV). The fact that only patients eligible for NAC were included in this study led to a selection of patients in advanced stages, and therefore perhaps also to a selection of patients who already had a high rate of sarcopenia (60%). The low heterogeneity of patients with regard to stage may lead to a narrower range of values for SMI, making it impossible to demonstrate its involvement in survival.

To our knowledge, we have identified only one article reporting body composition before and after three cycles of NAC in patients with EOC (Rutten et al., 2016). Compared with our study, we might note a difference in patient selection, since we only included patients eligible for interval surgery. The authors identified muscle loss and visceral fat loss as factors negatively related to overall survival. In line with this study, our results report a link between fat loss, particularly visceral fat loss, and overall survival. It is difficult to determine whether fat loss is associated with tumor progression, the effect of chemotherapy or some other mechanism. However, in our study, the favorable evolution of the tumor under chemotherapy, which makes it possible to space out surgical interventions, is not linked to these changes in body fat (Supplementary Table 1). Without ruling out the possibility of chemotherapy-related toxicity, we may wonder why the loss of body fat occurs mainly at the visceral level. In the event of a reduction in food intake, it has been shown that, physiologically, caloric restriction preferentially reduces visceral over subcutaneous fat (Janssen and Ross, 1999). Nevertheless, it cannot be ruled out that the particular localization of ovarian cancer in the abdominal cavity may favour privileged interactions between these cancer cells and abdominal adipose tissue. It constitutes a source of energy (by delipidation of adipocytes) and a metabolic/cytokinic support for their proliferation and local and distant implantation (Motohara et al., 2019). Moreover, the proximity of ovarian tumour cells to adipocytes may induce a metabolic shift towards fatty acid consumption and beta-oxidation, which may be associated with tumour progression (Chen et al., 2019; Wang et al., 2017).

The prognostic significance of adiposity is still debated. Our results indicate that obese patients are particularly affected by fat and lean mass loss. Nevertheless, there is no link between BMI and survival (RFS and OS). This ambiguity has already been noted in the literature. In 2023, Cuello’s publication showed that high visceral fat mass and metabolic dysfunction, not BMI, are associated with poorer survival in patients with EOC (Cuello et al., 2023). Furthermore, our results showed that patients with poorer survival had a rapid and severe decrease in body fat (over 100 days, −28% for VATI and −18% for WFM), suggesting specific metabolic changes and a strong energy imbalance. Some hypotheses can be put forward: i/an increase in inflammatory processes that could lead to a greater reduction in food intake, placing these patients in an energy deficit (Martin et al., 2021). Measures of inflammation and caloric intake should therefore be evaluated in future studies; ii/a decrease in food intake could be coupled with hypermetabolism leading to rapid loss of adipose tissue. Although never demonstrated in ovarian cancer, hypermetabolism has already been measured in cancer patients, affecting over a third of patients and having a major impact on patient survival (Vazeille et al., 2017). To manage these patients, who are rapidly losing body fat and whose risk of death is particularly high, the literature suggests two options: either nutritional supplementation to compensate for the energy deficit (Cotogni et al., 2022; Jouinot et al., 2020) or nutritional or drug management to rebalance lipid metabolism (Cuello et al., 2023). Only the identification of the mechanism(s) responsible for fat loss, using simple, validated markers or biomarkers, can guide the clinician in the choice of nutritional or medicinal support.

One of the strengths of our work is the homogeneity and representativeness of our cohort, since all patients had advanced stage III-IV FIGO EOC, and were treated with the same drugs and protocol at the same center. However, our study also has certain limitations, including its retrospective nature and the fact that our sample size (patients with NAC) decreased considerably, which limited the power of statistical analyses. We acknowledge that the small sample size, particularly the limited number of obese patients (N = 8), may have reduced the reliability of subgroup analyses. While our findings suggest that obese patients are particularly susceptible to fat and muscle loss during NAC, these results should be interpreted with caution. Future multicenter studies with larger cohorts are needed to validate these observations and to further explore the impact of NAC on body composition parameters and prognostic implications. Nutritional supplementation and pharmacological management of lipid metabolism represent promising strategies to mitigate fat loss and improve survival outcomes. However, the efficacy of these interventions must be validated in prospective clinical trials.

In conclusion, our study highlights the high prevalence of sarcopenia in women with advanced ovarian cancer and identifies rapid and severe loss of visceral fat as a prognostic factor for poorer OS. These findings suggest the potential of early nutritional and/or pharmacological interventions to promote muscle and adipose tissue maintenance and improve outcomes for women with advanced ovarian cancer. Future research should focus on identifying the mechanisms underlying fat loss and evaluating the efficacy of targeted interventions in this high-risk population.

## Data Availability

The original contributions presented in the study are included in the article/Supplementary Material, further inquiries can be directed to the corresponding author.
