# Supplementary material for: Adipose tissue loss during neoadjuvant chemotherapy: a key prognostic factor in advanced epithelial ovarian cancer
Source: Front Physiol. 2025 Mar 25;16:1537484. doi: 10.3389/fphys.2025.1537484 (PMC11977129; doi:10.3389/fphys.2025.1537484)
Supplement: Supplementary file 1 [file Table1.DOCX]

**Table 1. Patient characteristics (n=53)**

| **Clinical parameters** | **n (%)** | **Median [IQR]** |
| --- | --- | --- |
| **Age** (years) |  | 68 [62.5-73.0] |
| **Menopausal status** |  |  |
| Non menopausal patients | 8 (15%) |  |
| **BMI** (kg/m²) |  | 24.7 [21.9-27.9] |
| Underweight (≤ 18.5) | 3 (6%) |  |
| Normal weight (18.5-24.9) | 25 (47%) |  |
| Overweight (25-29.9) | 17 (32%) |  |
| Obese (≥30.0) | 8 (15%) |  |
| **BW loss during the past 2-6 months** (%) |  | 7.3 [4.1-10.6] |
| Patients who experienced BW loss | 17 (32%) |  |
| **Ascites punction** (L) |  | 3.8 [0.9-5.0] |
| Patients with ascites removed | 21 (40%) |  |
| **Histology** |  |  |
| Pure HGSOC | 43 (81%) |  |
| Other cancer types | 10 (19%) |  |
| **FIGO Stage** |  |  |
| Stage III | 35 (66%) |  |
| Stage IV | 14 (26%) |  |
| Unknown | 4 (8%) |  |
| **Treatment** |  |  |
| Surgery after 3-4 cycles of chemotherapy | 26 (49%) |  |
| Surgery after 6 cycles of chemotherapy | 10 (19%) |  |
| Not operable at anytime | 17 (32%) |  |
| **Follow up** (months) |  | 13.0 [9.0-22.8] |

BMI, Body Mass Index; BW, Body Weight; FIGO, Federation of Gynaecology and Obstetrics classification for staging ovarian cancer; HGSOC, high grade serous ovarian cancer; IQR, interquartile range; SD, standard deviation

**Table 2. Clinical and body composition parameters at baseline and their association with survival in ovarian cancer patients treated with NAC.**

| **Parameters** | **Groups** | **n (%)** | **Median [IQR]** | ***p* value for RFS**  (Low *vs.* High) | ***p* value for OS**  (Low *vs.* High) |
| --- | --- | --- | --- | --- | --- |
| **Clinical Parameters** | | | | | |
| Age (years) | All patients  Tertile 1 (Younger)  Tertile 3 (Older) | 53 (100%)  17 (32%)  20 (38%) | 68.0 [62.5-73.0]  51.0 [33.5-62.5]  75.0 [71.0-78.5] | 0.245 | 0.896 |
| BMI (kg/m²) | All patients  Under/Normal weight Overweight  Obese | 53 (100%)  28 (53%)  17 (32%)  8 (15%) | 24.7 [21.9-27.9]  22.0 [21.0-23.8]  27.0 [26.4-28.0]  32.5 [31.0-37.9] | 0.885 | 0.907 |
| **Muscle parameters** | | | | | |
| SMI (cm²/m²) | All patients  Sarcopenic patients (Low SMI)  Non-sarcopenic patients (High SMI) | 53 (100%)  32 (60%)  21 (40%) | 39.4 [35.4-44.2]  36.6 [34.1-38.8]  45.4 [43.1-50.6] | 0.234 | 0.830 |
| Estimated LBM (kg) | All patients  Tertile 1 (Low LBM)  Tertile 3 (High LBM) | 53 (100%)  17 (32%)  18 (34%) | 37.1 [33.3-40.5]  32.5 [30.7-33.4]  41.1 [40.5-43.0] | 0.764 | 0.949 |
| **Fat Parameters** | | | | | |
| VATI (cm²/m²) | All patients  Tertile 1 (Low VATI)  Tertile 3 (High VATI) | 53 (100%)  18 (34%)  18 (34%) | 22.5 [11.6-47.3]  9.0 [2.8-12.5]  68.0 [46.3-100.1] | 0.537 | 0.164 |
| SATI (cm²/m²) | All patients  Tertile 1 (Low SATI)  Tertile 3 (High SATI) | 53 (100%)  18 (34%)  18 (34%) | 66.3 [48.0-121.6]  34.7 [26.0-48.9]  125.6 [115.9-157.7] | 0.793 | 0.133 |
| Estimated WFM (kg) | All patients  Tertile 1 (Low WFM)  Tertile 3 (High WFM) | 53 (100%)  18 (34%)  18 (34%) | 21.3 [16.0-33.1]  12.7 [10.1-16.1]  36.2 [32.8-42.5] | 0.673 | 0.212 |

BMI, Body Mass Index; IQR, interquartile range; LBM, Lean Body Mass; OS, Overall Survival; RFS, Recurrence-Free Survival; SATI, Subcutaneous Adipose Tissue Index; SMI, Skeletal Muscle Index; VATI, Visceral Adipose Tissue Index; WFM, Whole-Body Fat Mass.

**Table 3.** **Changes in clinical parameters and body composition between C0 and C3 and their association with survival in ovarian cancer patients treated with NAC.**

| **Parameters** | **Groups** | **n (%)** | **Median [IQR]** | ***p* value for RFS**  (Stable/Gain *vs.* Loss) | ***p* value for OS**  (Stable/Gain *vs.* Loss) |
| --- | --- | --- | --- | --- | --- |
| **Clinical Parameters** | | | | | |
| Interval surgery at 3-4 cycles | Yes  No | 26 (49%)  27 (51%) |  | **0.013 *** | 0.336 |
| **Muscle parameters** | | | | | |
| SMI (%/100 days) | All patients  SMI Stable/Gain  SMI Loss | 53 (100%)  26 (49%)  27 (51%) | -2.2 [-8.4 ; +6.5]  +6.5 [+3.0 ; +9.0]  -7.8 [-12.8 ; -4.3] | 0.113 | 0.645 |
| Estimated LBM (%/100 days) | All patients  LBM Stable/Gain  LBM Loss | 53 (100%)  28 (53%)  25 (47%) | -1.8 [-6.9 ; +5.4]  +4.5 [+2.2 ; +7.6]  -6.9 [-12.3 ; -3.9] | 0.433 | 0.439 |
| **Fat Parameters** | | | | | |
| VATI (%/100 days) | All patients  VATI Stable/Gain  VATI Loss | 53 (100%)  19 (36%)  34 (64%) | -10.5 [-32.5 ; +6.6]  +17.2 [+5.0 ; +39.0]  -27.7 [-39.6 ; -10.8] | 0.249 | **0.031 *** |
| SATI (%/100 days) | All patients  SATI Stable/Gain  SATI Loss | 53 (100%)  16 (30%)  37 (70%) | -12.1 [-27.3 ; +1.4]  +10.3 [+2.4 ; +28.5]  -19.0 [-32.5 ; -10.6] | 0.075 | 0.079 |
| Estimated WFM (%/100 days) | All patients  WFM Stable/Gain  WFM Loss | 53 (100%)  18 (34%)  35 (66%) | -7.6 [-20.8 ; +2.0]  +4.9 [+1.4 ; +19.4]  -17.0 [-24.7 ; -7.6] | 0.269 | **0.046 *** |

C0, at diagnosis (before initiation of NAC); C3, after completion of 3 to 4 cycles of NAC (interval); IQR, interquartile range; LBM, Lean Body Mass; OS, Overall Survival; RFS, Recurrence-Free Survival; SATI, Subcutaneous Adipose Tissue Index; SMI, Skeletal Muscle Index; VATI, Visceral Adipose Tissue Index; WFM, Whole-Body Fat Mass.

**Table S1. Characteristics of patients with and without interval surgery after 3-4 cycles of chemotherapy.**

| **Parameters** | **Surgery after 3-4 cycles of chemotherapy** | | ***p* value**  (Yes *vs.* No) |
| --- | --- | --- | --- |
|  | Yes (n=26) - Median [IQR] | No (n=27) - Median [IQR] |  |
| **Clinical parameters at baseline** | | | |
| Age (years) | 68.0 [64.5 ; 72.0] | 68.0 [61.0 ; 75.0] | 0.733 |
| BMI (kg/cm²)  BMI categories  Underweight / Normal weight  Overweight  Obese | 25.0 [21.4 ; 27.9]  13 (50)  10 (38)  3 (12) | 24.7 [23.0 ; 27.7]  15 (55.5)  7 (26)  5 (18.5) | 0.863  0.521 |
| Histotype of tumor  Pure HGSOC  Other cancer types | 21 (81)  5 (19) | 22 (81)  5 (19) | >0.999 |
| Patients with ascites removed | 9 (35) | 12 (44) | 0.577 |
| **Body composition parameters at baseline** | | | |
| SMI (cm²/m²)  Sarcopenic patients | 40.6 [35.4 ; 43.4]  15 (58) | 38.8 [35.3 ; 45.1]  17 (63) | 0.923  0.782 |
| Estimated LBM (kg) | 37.2 [33.4 ; 38.9] | 36.1 [32.9 ; 40.8] | 0.936 |
| VATI (cm²/m²) | 22.3 [11.8 ; 45.2] | 23.0 [11.4 ; 48.9] | 0.983 |
| SATI (cm²/m²) | 69.8 [45.8 ; 124.2] | 66.3 [48.7 ; 80.0] | 0.247 |
| Estimated WFM (kg) | 21.0 [14.6 ; 35.2] | 21.5 [16.1 ; 31.9] | 0.463 |
| **Change in body composition parameters between C0 and C3** | | | |
| SMI (%/100 days) | -2.8 [-9.2 ; +7.6] | -0.1 [-7.2 ; +6.5] | 0.689 |
| Estimated LBM (%/100 days) | -2.3 [-7.5 ; +6.5] | -0.1 [-6.1 ; +5.3] | 0.683 |
| VATI (%/100 days) | -9.8 [-25.2 ; +7.5] | -19.3 [-34.0 ; +2.7] | 0.436 |
| SATI (%/100 days) | -7.9 [-27.2 ; +6.5] | -15.0 [-27.6 ; -5.4] | 0.310 |
| Estimated WFM (%/100 days) | -5.2 [-17.8 ; +4.0] | -11.2 [-23.8 ; -0.8] | 0.199 |

BMI, Body Mass Index; C0, at diagnosis (before initiation of NAC); C3, after completion of 3 to 4 cycles of NAC (interval); HGSOC, high grade serous ovarian cancer; IQR, interquartile range; LBM, Lean Body Mass; SATI, Subcutaneous Adipose Tissue Index; SMI, Skeletal Muscle Index; VATI, Visceral Adipose Tissue Index; WFM, Whole-Body Fat Mass.
